# Pigs Immunized with the Virus-like Particle Vaccine Are Protected against the Hepatitis E-3 Virus

**DOI:** 10.3390/vaccines9111265

**Published:** 2021-11-02

**Authors:** Hyeon-Jeong Go, Byung-Joo Park, Hee-Seop Ahn, Dong-Hwi Kim, Da-Yoon Kim, Jae-Hyeong Kim, Joong-Bok Lee, Seung-Yong Park, Chang-Seon Song, Sang-Won Lee, Yang-Kyu Choi, In-Soo Choi

**Affiliations:** 1 Department of Infectious Disease, College of Veterinary Medicine, Konkuk University, 120 Neundong-ro, Gwangjin-gu, Seoul 05029, Korea; goluffy@konkuk.ac.kr (H.-J.G.); twilight@konkuk.ac.kr (B.-J.P.); frequency0@konkuk.ac.kr (H.-S.A.); opeean0@konkuk.ac.kr (D.-H.K.); kimda68@konkuk.ac.kr (D.-Y.K.); mirine2u@konkuk.ac.kr (J.-H.K.); virus@konkuk.ac.kr (J.-B.L.); paseyo@konkuk.ac.kr (S.-Y.P.); songcs@konkuk.ac.kr (C.-S.S.); odssey@konkuk.ac.kr (S.-W.L.); 2 Department of Laboratory Animal Medicine, College of Veterinary Medicine, Konkuk University, 120 Neundong-ro, Gwangjin-gu, Seoul 05029, Korea; yangkyuc@konkuk.ac.kr

**Keywords:** Hepatitis E virus, virus-like particle, 239 amino acids, viremia, fecal viral shedding, liver fibrosis, baculovirus expression system

## Abstract

In this study, we generated the HEV virus-like particle (VLP) vaccine expressing 239 amino acids (367–605 aa) of the HEV-3 ORF2 using the baculovirus expression system. The HEV-3-239-VLP vaccine efficacy was evaluated by dividing 12 pathogen-free pigs into four groups: negative control, positive control, 100 μg VLP-, and 200 μg VLP-vaccinated groups for 10 weeks. The pigs in either of the vaccinated groups were administered the corresponding first and booster doses on weeks 0 and 2. At week 4, the positive control and two vaccinated groups were challenged with 10^6^ HEV-3 genomic equivalent copies; viremia and fecal shedding of the virus were identified in pigs in the positive control and 100 μg VLP-vaccinated pigs showed transient viremia and fecal viral shedding. However, no viruses were detected in the serum or fecal samples of the 200 μg VLP-vaccinated pigs. The 100 and 200 μg VLP-vaccinated pigs had significantly higher (*p* < 0.01) anti-HEV antibodies than the negative control pigs from weeks 6–10 with normal levels of liver enzymes. The 200 μg VLP-vaccinated pigs showed statistically less liver tissue fibrosis (*p* < 0.05) than that of the positive control pigs. Thus, the novel baculovirus expression system-generated VLP vaccine dose-dependently protects against HEV-3 challenge and may be useful in other animal species, including humans.

## 1. Introduction

Hepatitis E virus (HEV), belonging to the genus Orthohepevirus of the family Hepeviridae, is a non-enveloped virus ranging from 27–34 nm [1]. It is classified into seven genotypes and divided into at least 31 subtypes. While HEV-1, -2, -3, -4, and -7 infect humans [2], HEV-1 and -2 infections are limited exclusively to humans and associated with the consumption of contaminated drinking water, mostly in developing countries [3]. The HEV-3 and 4 are zoonotically transmitted by eating uncooked pork in developed countries [4,5]. In particular, HEV-3, and -4 can lead to chronic hepatitis in immunocompromised patients [6].

HEV has a positive-sense single-stranded RNA genome of approximately 7.2 kb length which consists of three open reading frames (ORFs). ORF1 encodes non-structural proteins essential for the viral life cycles, ORF2 encodes a viral capsid protein, and the ORF3 protein is associated with releasing infectious viral particles [7,8,9]. Among the viral proteins, the capsid protein recognized as the main antigen induces neutralizing antibodies. Hence, the capsid protein is generally used for vaccine development [10]. Owing to the difficulty of cultivating HEV in cell culture systems, studies have focused on the virus-like particle (VLP) composed of the capsid protein for developing vaccines.

VLP vaccines have been generated with *Escherichia coli* and the baculovirus expression systems [11,12,13]. Furthermore, VLPs have been manufactured by expressing different sizes of capsid proteins, and their efficacies have been evaluated. Recently, three types of VLPs with different sizes of the capsid protein were generated from *E. coli* as follows: p179 (439–617 aa), p239 (368–606 aa), and p496 (112–606 aa) [11,13,14]. Among them, the p239 VLP has been found to induce neutralizing antibodies [15]. In the preclinical assessment and phase III clinical trial, the p239 VLP has been verified as highly immunogenic and safe [16,17]. The p239 VLP was finally approved as an official HEV vaccine in China in October 2012 [18].

The baculovirus expression system has the advantage of producing VLP particles that are morphologically and antigenically similar to the native virus particles [19]. The N-terminal truncation of the ORF2 capsid protein of HEV is essential for the VLP assembly in the baculovirus system [20]. Expression of the partial capsid protein with 112–660 amino acids using baculovirus system produced 50 kDa T = 1 symmetry HEV VLP [20]. The HEV VLP can induce seroconversion and prevent HEV infection in cynomolgus monkeys [21]. Therefore, the baculovirus system is recognized as a favorable platform for producing VLP vaccine candidates.

In this study, a VLP vaccine made up of 239 aa of HEV-3 was produced by the baculovirus expression system. The vaccine was sufficient for protecting pigs from HEV challenges. This HEV VLP vaccine, generated from a eukaryotic expression system, is expected to be applicable for preventing HEV infections.

## 2. Materials and Methods

### 2.1. Selection of the HEV-3-239 Capsid Antigen and Producing Recombinant Baculovirus

Sequences of HEV-3 isolated from a pig in Korea (Gene bank No.: FJ426404.1) for vaccine production were obtained from the National Center for Biotechnology (NCBI). The amino acid sequences of HEV-3 corresponding to p239 (368–606 a.a) of HEV-1 (Gene bank No.: L08816.1) were used to produce recombinant baculovirus. Codon optimization of HEV ORF2 239 aa gene was optimized for using a baculovirus system. The amplified gene was ligated to the pFastbac™ vector and transformed into the competent cells. After the sequence analysis of the inserted gene, the pFastbac™-HEV-3-239 was transformed to the DH10bac™ via heat shock. After isolating bacmid-HEV-3-239 from DH10bac™, the transfecting sf9 cells were carried out. The transfected sf9 cells were incubated for 72 h at 27 °C and the rates of cytopathic effect (CPE) were measured. The first passage (P1) of recombinant baculoviruses were harvested from the cells.

### 2.2. Purification of HEV-239 VLP

The second passage (P2) was performed by amplifying the first recombinant baculovirus. It was used to produce HEV-3-239-VLP. The recombinant baculovirus-infected sf9 cells were burst by repeated freezing and thawing ultrasound with 20% amplitude for obtaining HEV-3-239-VLPs inside the sf9 cells. After removing the sf9 cell debris with high centrifugation (6000× *g*, 30 min), a 0.22 μm filter was used to remove the sf9 cell debris and baculoviruses. The collected proteins were concentrated by ultracentrifugation (51,000× *g*, 1 h 30 min) to obtain a protein pellet. After dissolving the protein pellet in the TNE buffer, the HEV-3-239-VLPs were purified by discontinuous sucrose gradient using ultracentrifugation (245,000× *g*, 2 h 30 min). The HEV-3-239-VLPs obtained from the visible protein band were dialyzed in 20 mM ammonium bicarbonate buffer. The capsid proteins which were not assembled into VLP were removed through dialysis.

### 2.3. Western Blot Analysis

The protein concentration of the purified recombinant rabbit HEV whole capsid protein [22] and HEV-3-239-VLP was measured using the Pierce™ BCA protein assay kit (ThermoFisher Scientific, Agawam, MA, USA). The proteins were loaded onto Tris-Glycine PAG 10–20% gel (LABISKOMA, Seoul, Korea) and transferred onto the nitrocellulose membrane. The membrane was blocked with 5% skimmed milk in PBS-T overnight at 4 °C and then incubated with the primary antibody, anti-Hepatitis E virus antibody, aa 434–457, clone 1E6 (Sigma-Aldrich, Burlington, MA, USA) for 1 h 30 min at 25 °C. The membrane was incubated with a secondary antibody for 1 h at 25 °C. Finally, the membrane was developed using the Supersignal West Pico Plus Chemiluminescent Substrate (ThermoFisher Scientific, Waltham, MA, USA).

### 2.4. Electron Microscopy

The purified HEV-3-239-VLPs were loaded onto formvar/carbon 200 mesh copper grid (TED PELLA, INC, Redding, CA, USA) and incubated for 5 min at room temperature, then washed with PBS and the excess VLP sample was removed using filter paper. After blocking with 1% BSA in PBS for 5 min, the VLP samples were incubated with the diluted primary antibody, anti-Hepatitis E virus polyclonal antibody from rabbit serum (1:50) on the surface of the grid for 30 min. The grid was washed with PBS six times and then incubated with anti-rabbit IgG-gold antibody produced in goat (Sigma-Aldrich, USA) at room temperature for 30 min. The grid was again washed six times with PBS, fixed in 4% paraformaldehyde, and negatively stained with 1% uranyl acetate solution. The stained VLP sample was scanned using a Tecnai G2 Spirit Twin transmission electron microscope at Korea Basic Science Institute (KBSI, Ochang, Korea).

### 2.5. Animals, Immunization, and Challenge Schedule

Twelve 7-week-old, female SPF miniature pigs were purchased through OPTIPHARM (Cheong-Ju, Korea). The experiment was conducted at Cronex (Cheong-Ju, Korea, CRONEX-IACUC: 202006004). The absence of HEV RNA and anti-HEV antibody in pig serum and fecal samples was verified through nested RT-PCR (Reverse Transcription PCR) and ELISA (Enzyme-Linked Immunosorbent Assay). The pigs were divided into four groups with three pigs in each group for the experiment: negative control, positive control (only HEV-challenged), 100 μg-vaccinated, and 200 μg-vaccinated groups. The serum and feces were collected from pigs every week. About 2 mg of aluminum hydroxide was used for each dose of the HEV-3-239-VLP vaccine. The time to start the experiment was set as week 0, the first inoculation of VLP vaccine was performed at week 0 and week 2, and the challenge of swine HEV-3 (10^6^ HEV copies/dose) was performed at week 4. The HEV-3-239 VLP vaccine was administered intramuscularly, and the swine HEV-3 was administered intravenously for the challenge. All pigs were euthanized with potassium chloride after administration with zolazepam at week 10 (W10).

### 2.6. HEV RNA, Anti-HEV IgG Antibody Analysis 

The blood collected from the pigs was centrifuged to separate the serum (2000× *g*, 20 min). The feces were dissolved in PBS at a ratio of 1:10 and the undissolved fecal mass was released by vortex mixer. The fecal suspension was centrifuged to separate the supernatant containing HEV (2000× *g*, 20 min). The HEV RNA was extracted from the collected serum and fecal supernatant using Patho Gene–spin™ DNA/RNA Extraction (iNtRON, Seongnam, Korea). The nested RT-PCR was performed using two pairs of primers [23] and Maxime RT-PCR PreMix and Maxime™ PCR PreMix (i-StarTaq) (iNtRON, Seongnam, Korea). The anti-HEV antibody titers were measured using WANTAI HEV-Ab ELISA (Beijing Wantai Biological, Beijing, China) according to the user’s instructions.

### 2.7. Examination of ALT, AST in the Porcine Serum

The serum ALT and AST values were measured at the NEODIN Biovet Laboratory (Korea) using a chemistry analyzer BS490 (Mindray, Shenzhen, China). The tests were repeated twice with serum, and the average value was used. 

### 2.8. Histopathology

After preparing a paraffin block from the liver tissue, the Hematoxylin and Eosin staining (H&E staining), and Masson′s trichrome staining were performed to confirm the overall structure and connective tissue of the liver. Observing the H&E-stained slides, the infiltration of inflammatory cells in the liver lobule was analyzed. To evaluate the proliferation of the connective tissue, all the trichrome-stained slides were observed at 100× magnification. The total area of the liver tissue and the area of the connective tissue was measured using MetaMorph^®^ (Molecular Devices, San Jose, CA, USA), and the ratio of the area of connective tissue to the total area of liver tissue was calculated.

### 2.9. Statistical Analysis

GraphPad Prism software (Version 8.0.2; GraphPad Software, San Diego, CA, USA) was used for the statistical analysis and the graphs were generated. All data were expressed as the means ± SDs of three replicates. The data were analyzed by repeated-measures ANOVA, two-way ANOVA, or one-way ANOVA. Dunnett’s multiple comparison test was carried out as the post hoc test. The statistical significance for each test was set at *p* < 0.05.

## 3. Results

### 3.1. Icosahedral Shaped, 20–30 nm-Sized, HEV-3-239-VLPs Was Assembled

**Figure 1:**
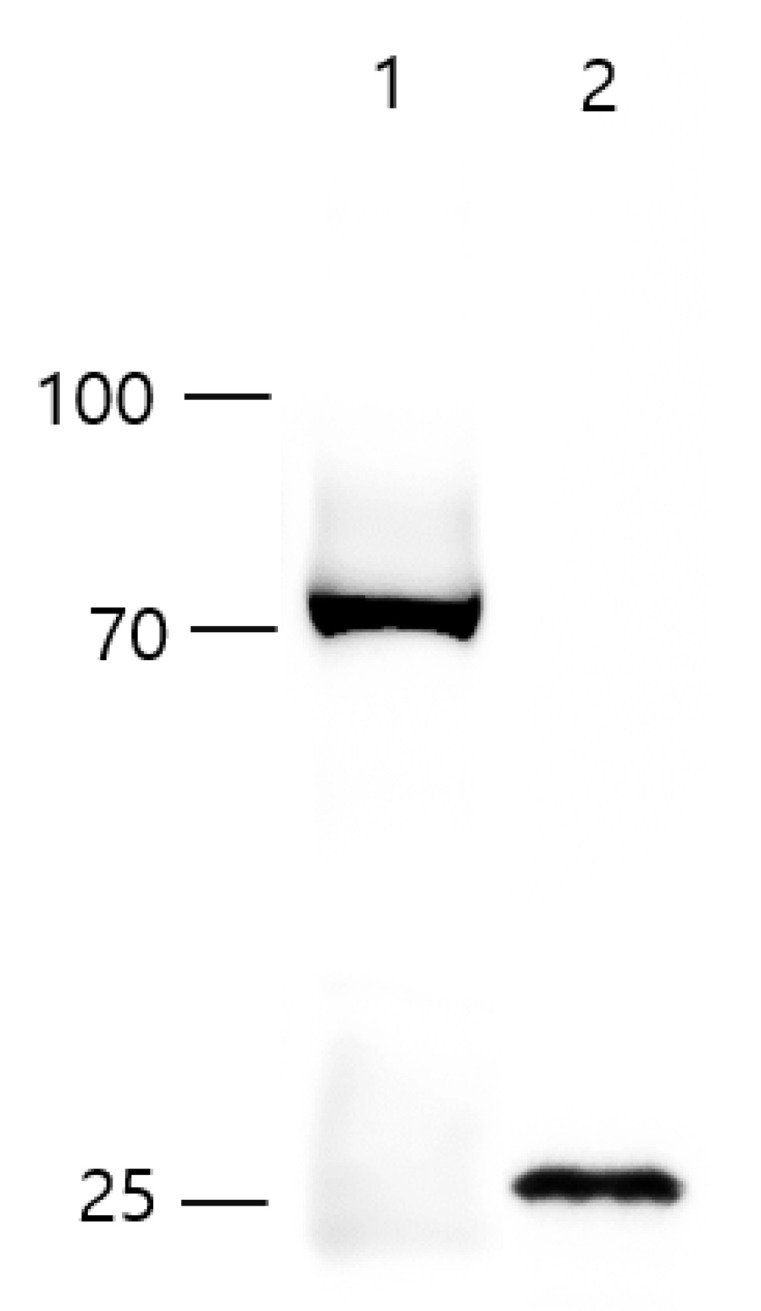
Molecular weight of the purified HEV-3-239-VLPs. The recombinant rabbit HEV whole capsid protein expressed by the *E. coli* system has a molecular weight of 72 kDa (lane 1). The monomer of HEV-3-239-VLP expressed in the baculovirus system has a molecular weight of 26 kDa (lane 2).

**Figure 2:**
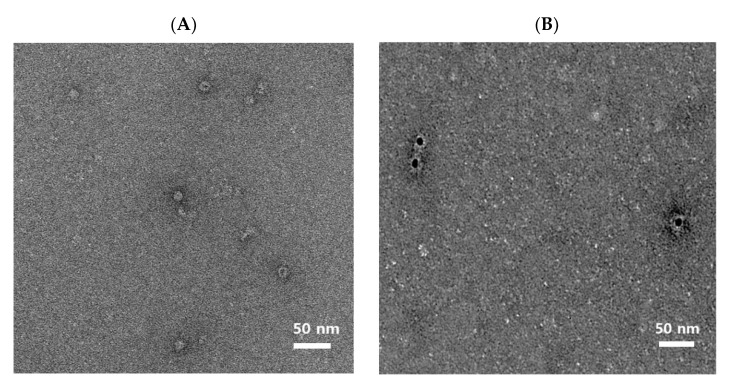
Electron microscopy images of the assembled HEV-3-239-VLPs. (**A**) Electron micrographs of negative stained HEV-3-239 VLPs. The diameter of the assembled VLP is 20–30 nm; (**B**) electron micrographs of the immunostained HEV-3-239 VLP. Gold particles with a size of 10 nm were attached to the VLPs.

Western blot analysis was performed with an anti-HEV monoclonal antibody to check the molecular weight of the purified HEV-3-239-VLPs. The proteins were decomposed into monomers by boiling them for 10 min. A 72 kDa-sized recombinant rabbit HEV whole capsid protein produced by *E. coli* was used as a control (Figure 1, lane 1). The HEV-3-239-VLP was identified as 26 kDa-sized monomers as expected (Figure 1, lane 2). After the HEV-3-239-VLP loaded grid was negatively stained, the shape and size of the assembled VLPs were observed with TEM. The HEV-3-239-VLPs were found to have an icosahedral shape with 20–30 nm diameter (Figure 2A). Immunostaining was performed with a gold particle-conjugated antibody, 10 nm-sized gold particles were attached to the surfaces of HEV-3-239-VLPs (Figure 2B). Therefore, it was confirmed that the predicted size of the capsid protein of HEV-3 was expressed in the baculovirus system, and the assembled HEV-3-239-VLP, with a diameter of 20–30 nm, was identified.

### 3.2. Fecal Viral Shedding and Viremia Were Not Detected in the Pigs Vaccinated with 200 μg of VLPs

The first vaccine inoculation was administered at week 0, and the booster shot was administered at week 2 in both the vaccinated groups. At week 4, the vaccinated group and the positive control group were challenged with the swine HEV-3. At week 10, the pigs were euthanized, and the livers were collected. The RNA of HEV-3 failed to be detected in all the sera and fecal samples collected every week of the experiment from pigs in the negative control group, as expected. In contrast, the pigs in the positive control group showed viremia from weeks 6 to 9, and fecal viral shedding from weeks 6 to 10 (Table 1). The HEV RNA was detected in the sera and feces of all three pigs in the positive control group. In contrast, transient viremia was detected in the sera of two out of three pigs at week 7, and fecal viral shedding was identified in two pigs at week 7 and one pig at week 8, out of three pigs vaccinated with 100 μg VLP. However, no viremia or fecal viral shedding were demonstrated during the whole experimental period in all the three pigs vaccinated with 200 μg of VLPs (Table 1). These results indicate that the dose of 200 μg of VLPs is sufficient for the protection of HEV-3 infection in pigs.

### 3.3. Vaccination with the HEV-3-239-VLP Induced the Anti-HEV Antibodies

**Figure 3:**
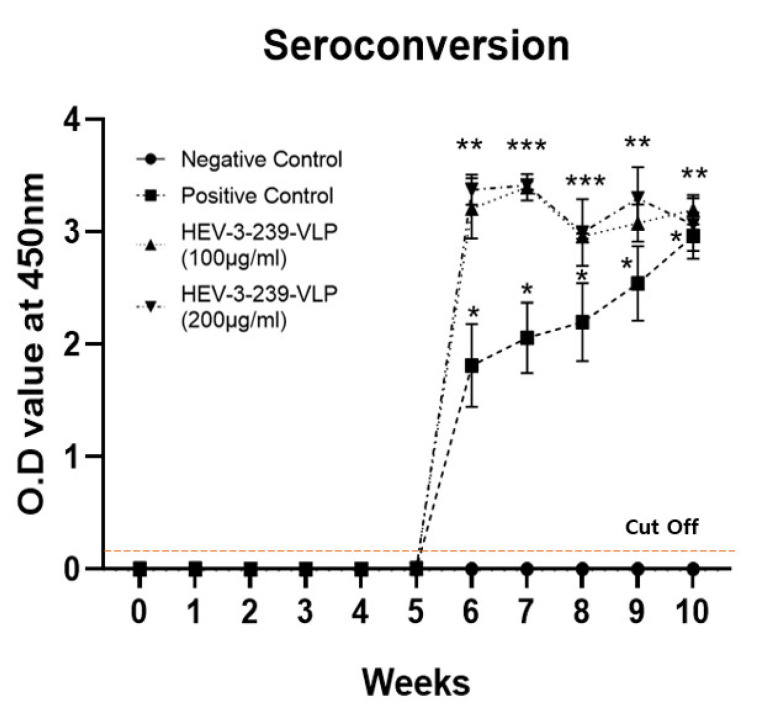
Seroconversion in pigs immunized with HEV-3-239 VLP. In the positive control group, the titer of the anti-HEV antibody increased relatively slowly after the challenge at W 4. In contrast, the titer of anti-HEV antibody in both the vaccinated groups significantly increased after 2 weeks of the viral challenge and was maintained for 5 weeks until the end of the experiment. Statistical analysis was conducted between the vaccinated pigs and negative ones and between the positive and negative control pigs. * *p* < 0.05, ** *p* < 0.01, *** *p* < 0.001.

The anti-HEV antibodies were not found in all the pigs in the negative control group in the entire experimental period, as expected. In the pigs in the positive control group, the anti-HEV antibody appeared at week 6 and slowly increased until week 10. Even though the antibody titers of the positive control pigs were significantly higher than those of the negative control ones, their titers were relatively lower than those of the vaccinated ones (Figure 3). The anti-HEV antibody titers of pigs in the two vaccinated groups were significantly (*p* < 0.01) higher from weeks 6 to 10 when compared with the negative control pigs (Figure 3). There were no differences in the antibody titers between the pigs vaccinated with 100 and 200 μg VLPs. However, their antibody titers were significantly higher than those of the positive control pigs. These results indicate that the two different doses of the VLP vaccine have enough immunogenicity for producing the anti-HEV antibodies. 

### 3.4. HEV-3-239 VLP Prevents Hepatitis 

**Figure 4:**
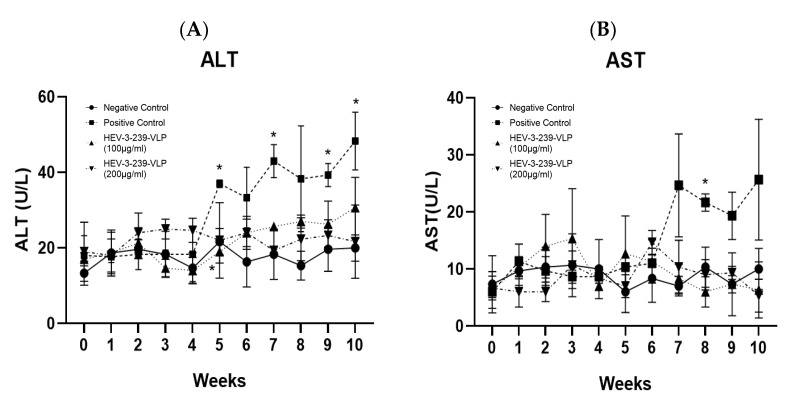
ALT value in the serum from all the pigs every week. (**A**) The ALT value of the negative control group was maintained at around 20 U/L. In contrast, the ALT value of the positive control group started to increase significantly at week 5 and exceeded the normal range (8.18–40.29) at week 7. The ALT value of the two vaccinated groups was maintained similar to the ALT value of the negative control group. (**B**) AST value in serum from all pigs for every week. The AST value of the negative control group was maintained at around 10 U/L. In contrast, the AST value of the positive control group started increasing at week 7 and remained relatively high compared to the values of other groups. The AST value of the two vaccinated groups was maintained similar to the AST value of the negative control group. The statistical analysis was conducted between the positive and negative control pigs and the vaccinated pigs and the negative ones. * *p* < 0.05.

Alanine aminotransferase (ALT) and aspartate aminotransferase (AST) are indicators of liver damage. The ALT values of the negative control group were maintained at around 20 U/L throughout the experiment. On the other hand, the ALT values of the positive control pigs were significantly higher (*p* < 0.05) than those of the negative control pigs at weeks 5, 7, 9, and 10. In particular, the ALT values exceeded the normal range (8.18–40.29) at weeks 7 and 10. The ALT values of the pigs in the two vaccinated groups were maintained at the normal ranges (Figure 4A). The AST value of the negative control pigs was maintained at around 10 U/L throughout the experiment. In contrast, the AST levels of pigs in the positive control group were significantly higher (*p* < 0.05) than those of negative control pigs at week 7 (Figure 4B). However, the AST levels of the positive control pigs also remained within the normal range (13–168). The AST values of the pigs in the two vaccinated groups were maintained similarly with the values of the negative control pigs. These results indicate that the VLP vaccine could prevent liver damage induced by HEV-3 infections.

### 3.5. Histopathological Analysis Indicates the Inhibition of Fibrosis in the Livers of Pigs Vaccinated with VLPs

**Figure 5:**
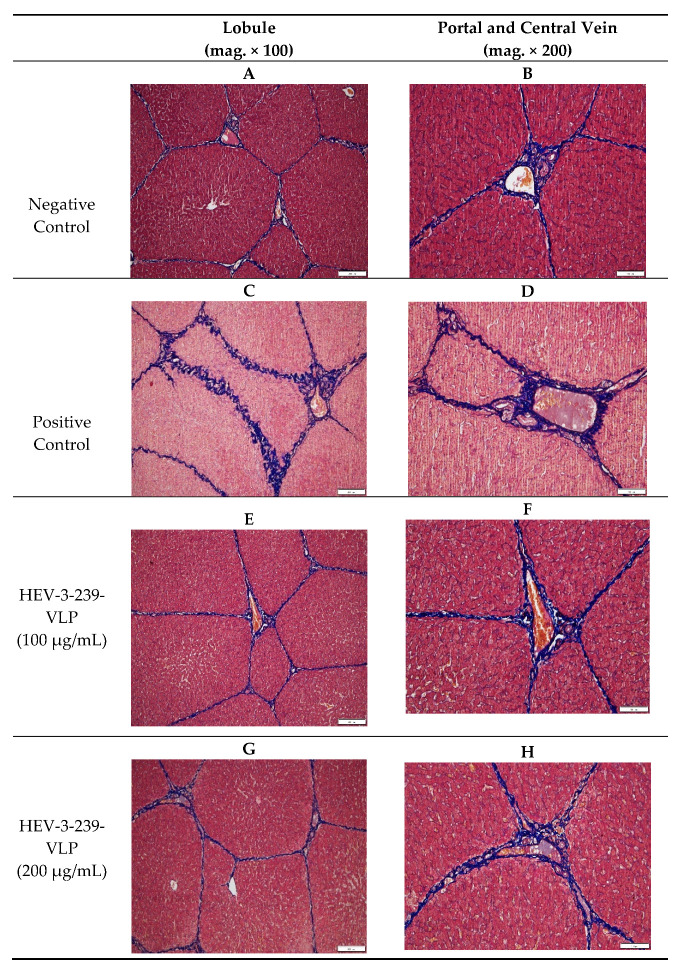
Lobules and portal and central veins of the liver tissues stained by the Masson’s trichrome staining method: (**A**,**B**) Relatively small amounts of blue-stained connective tissues between the lobules and around the portal veins of the negative control pigs; (**C**,**D**) Excessive connective tissues between the lobules and around central veins of the positive control pigs; (**E**,**F**), smaller connective tissues between the lobules and around the portal veins of pigs vaccinated with 100 μg of VLP vaccine than those identified in the positive control pigs. (**G**,**H**) The smaller connective tissues between lobules and around the portal veins of the pigs vaccinated with 200 μg of VLP vaccine than those in the positive control pigs.

**Figure 6:**
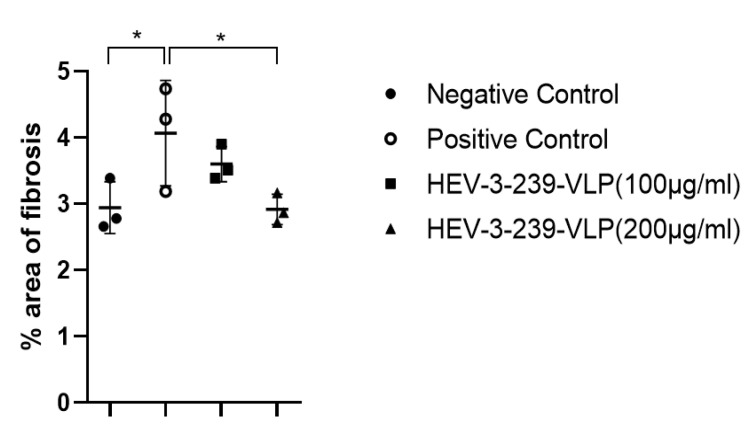
Percentage of the area of fibrosis in the Masson’s trichrome-stained liver tissue slides. In the positive control group, the blue-stained connective tissue was significantly proliferated by HEV infection compared to other groups. The proliferation of liver connective tissue caused by HEV infection was prevented by the HEV-3-239 VLP vaccination. Statistical analysis was conducted between the negative control pigs and positive control ones and between the positive control pigs and vaccinated ones. * *p* < 0.05.

The liver tissues of pigs in the negative control group showed small numbers of mononuclear cells and fibroblast in the interlobular connective tissue and around portal triad regions. However, the liver tissue of pigs in the positive control showed more focal infiltration of the mononuclear cells in the intralobular region and fibroblasts between lobules than that in negative control pigs (data not shown). Pigs in both the vaccinated groups also showed smaller numbers of mononuclear cells and fibroblasts than those of the positive control pigs in the liver tissues (data not shown). The degree of fibrosis in the liver tissues was determined by staining with Masson’s trichrome. Fibrosis was mainly found in the interlobular connective tissue and in the portal and central veins of the livers of pigs which were in the HEV-infected positive control group (Figure 5C,D). The degree of fibrosis of pigs vaccinated with 100 and 200 μg of VLPs seemed to be similar to that found in the negative control pigs (Figure 5A,B,E,F). Therefore, the actual degrees of fibrosis were calculated by comparing the areas of fibrosis to the total liver tissue areas. Fibrosis of the liver tissues of the positive control pigs was significantly higher (*p* < 0.05) than that of the negative control ones (Figure 6). However, the degree of fibrosis of the liver tissues of pigs vaccinated with 200 μg of VLP was significantly lower (*p* < 0.05) than that of the positive control pigs. Although the fibrosis of the liver tissues of the pigs vaccinated with 100 μg of VLP was smaller than that of the positive control pigs, there were no statistical differences between the two groups (Figure 6). These results indicate that the VLP vaccine could prevent the fibrosis induced in the liver tissues of the pigs infected with HEV-3.

## 4. Discussion

The HEV infection is mostly asymptomatic, but can lead to acute hepatitis [24]. HEV is largely responsible for 50% of acute viral hepatitis in the endemic region [25]. The mortality rate by HEV infection usually ranges from 0.5 to 4% [26] but can increase to 20% with the preterm delivery in pregnant women in developing countries [27,28]. In addition, outbreaks of autochthonous and sporadic acute hepatitis were reported due to zoonotic transmission of HEV such as consuming undercooked meat in developed countries [29]. In particular, HEV is considered to be triggered by chronic hepatitis in immunocompromised patients in developed countries [6]. Acute hepatitis caused by HEV infection can be treated using antiviral drugs such as ribavirin, but the potential is limited due to the inability of the antiviral drugs to be administered to high-risk patients such as pregnant women [30]. Preventing HEV infection in advance is the most important thing, but so far, no other HEV vaccine has been approved except the one licensed in China [16,31]. This necessitates the development of more HEV vaccines.

We developed the HEV VLP vaccine with 239 aa of HEV capsid protein using the baculovirus system. The p239 (368–606 aa), which is 92 aa extension from the N-terminus of the E2 domain can self-assemble into VLP form [15]. The p239 VLP has already been reported to possess high immunogenicity and antigenicity, and 100% of high efficacy as a vaccine through preclinical studies and clinical trials, and has been officially approved in China [15,16,31]. The recombinant protein vaccine produced using the baculovirus system based on p495 was known to have an efficacy of 95.5% in phase I clinical trials, but additional clinical trials were not conducted. There has also been no attempt to develop a VLP vaccine by expressing a protein smaller than p495 [12]. Therefore, in this study, the HEV-3-239-VLP vaccine was developed by expressing the highly immunogenic p239 of the swine HEV-3 capsid protein using the baculovirus system. The high efficacy of the developed HEV-3-239-VLP vaccine against HEV-3 was confirmed by immunizing the pigs.

Titers of anti-HEV antibodies in all the pigs inoculated with 100 and 200 μg of HEV-3-239-VLP were significantly increased (*p* < 0.01) 2 weeks after the HEV-3 challenge. At week 7, two out of three pigs showed transient viremia and two pigs at week 7 and one pig at week 8 out of three pigs vaccinated with 100 μg of VLPs showed fecal viral shedding. However, all the pigs vaccinated with 200 μg of VLPs showed no viremia and fecal viral shedding for entire experiment periods. Unlike the results of this study, the HEV 239 vaccine made using the *E. coli* expression system induced a gradual increase in the anti-HEV antibodies by two doses of vaccine before the challenge in all the immunized monkeys. Similarly, when the monkeys vaccinated with 5 and 10 μg of HEV 239 vaccine were challenged with 10^7^ genomic doses of HEV-1, the fecal viral shedding was observed for 3 weeks and 1 week, respectively. However 20 μg of HEV 239 vaccine protected HEV infection against the same genomic dose of HEV-1 [15]. Therefore, the HEV-3-239-VLP vaccine showed dose-dependent protection against HEV infection, similar to the other HEV vaccines.

In this study, the ALT values of the positive control pigs were significantly (*p* < 0.01) elevated at weeks 5, 7, 9, 10 compared to those of the negative control pigs. In particular, at weeks 7 and 10, the ALT values of the positive control pigs were over 40 U/L, which is over the normal range (8.18–40.29 U/L). However, the ALT values of the two vaccinated groups of pigs did not elevate and were maintained within the normal ranges, similar to the ALT values of the negative control pigs. Although the AST values of all the groups of pigs ranged within the normal range (13–168 U/L), the AST value of the positive control pigs was significantly (*p* < 0.05) increased at week 7 compared to the AST values of the negative control pigs. According to the results of a study evaluating the efficacy of the p179 VLP vaccine made using the *E. coli* system in rabbits, significant ALT elevation was identified in three out of five rabbits of the unvaccinated control group inoculated with HEV-4. Additionally, in an experiment evaluating the efficacy of the p239 VLP vaccine, two out of three monkeys were reportedly infected with HEV-1 and all three monkeys infected with HEV-4 showed a significant increase in the ALT values. However, the ALT values of all the animals immunized with the p179 and p239 VLP vaccines did not increase [15,32]. Given that the HEV-3-239-VLP prevented an increase in ALT and AST levels, which are indicators of liver damage, in the vaccinated pigs, the HEV-3-239-VLP could be considered to prevent liver tissue damage caused by HEV infection.

In the histopathological analysis of the liver tissues of all the groups of pigs, the intralobular infiltration of the mononuclear cell was observed in the liver tissues of the positive control pigs (data not shown). However, in the liver tissues of the two vaccinated groups, there were relatively small amounts of mononuclear cells observed. The focal and multifocal mononuclear cell infiltration were similarly observed in the liver tissue of the HEV-4 infected rabbits [32]. Additionally, in the experiment conducted by inoculating the baculovirus-expressed 55kDa HEV-1 ORF2 protein into monkeys, large histopathological changes were identified in the monkeys of the placebo control group but minimal histopathological changes were observed in only one monkey of the low-dose vaccinated group [33]. Interestingly, this study verified significant fibrosis in the liver tissues of the pigs infected with HEV-3. Conversely, in the liver tissues of 200 μg of the VLP-vaccinated pigs, the degree of fibrosis was significantly lower than those of the positive control pigs. The previous study showed that fibrosis was detected in the liver tissues of the swine HEV and rabbit HEV-infected rabbits. Thus far, although there has been no attempt to confirm the degree of fibrosis of the liver tissues during the evaluation of the efficacy of the developed HEV vaccine, we suggest that fibrosis of the liver tissues has the potential to be used as a new indicator for evaluating the HEV infection and vaccine efficacy in the future. Thus, the developed HEV-3-239-VLP vaccine in this study would be used for preventing hepatitis in pigs and be used effectively to restrain liver fibrosis.

## 5. Conclusions

The HEV-3-239-VLP was developed with 239 aa of the swine HEV-3 capsid protein using the baculovirus system. The developed HEV VLP vaccine demonstrated dose-dependent protection from HEV-3 infection in pigs. This newly developed VLP vaccine would be applicable for preventing HEV infections in humans and other animal species.

## Figures and Tables

**Table 1:** The number of pigs with HEV RNA detected in feces and serum collected each week.

Number of Pigs HEV RNA Detected												
Group	Weeks Post-Inoculation											
	Sample	0	1	2	3	4	5	6	7	8	9	10
Negative Control	Serum	0/3	0/3	0/3	0/3	0/3	0/3	0/3	0/3	0/3	0/3	0/3
	Feces	0/3	0/3	0/3	0/3	0/3	0/3	0/3	0/3	0/3	0/3	0/3
Positive Control	Serum	0/3	0/3	0/3	0/3	0/3	0/3	1/3	2/3	1/3	2/3	0/3
	Feces	0/3	0/3	0/3	0/3	0/3	0/3	2/3	2/3	3/3	2/3	1/3
HEV-3-239-VLP (100 μg/mL)	Serum	0/3	0/3	0/3	0/3	0/3	0/3	0/3	2/3	0/3	0/3	0/3
	Feces	0/3	0/3	0/3	0/3	0/3	0/3	0/3	2/3	1/3	0/3	0/3
HEV-3-239-VLP (200 μg/mL)	Serum	0/3	0/3	0/3	0/3	0/3	0/3	0/3	0/3	0/3	0/3	0/3
	Feces	0/3	0/3	0/3	0/3	0/3	0/3	0/3	0/3	0/3	0/3	0/3
